# Boldness predicts an individual's position along an exploration–exploitation foraging trade‐off

**DOI:** 10.1111/1365-2656.12724

**Published:** 2017-07-24

**Authors:** Samantha C. Patrick, David Pinaud, Henri Weimerskirch

**Affiliations:** ^1^ School of Environmental Sciences University of Liverpool Liverpool UK; ^2^ Centre d'Etudes Biologiques de Chizé UMR 7372 CNRS—Université La Rochelle Villiers‐en‐Bois France

**Keywords:** albatrosses, area‐restricted search, first passage time, marginal value theorem, personality, seabirds

## Abstract

1. Individuals do not have complete information about the environment and therefore they face a trade‐off between gathering information (exploration) and gathering resources (exploitation). Studies have shown individual differences in components of this trade‐off but how stable these strategies are in a population and the intrinsic drivers of these differences is not well understood.
2. Top marine predators are expected to experience a particularly strong trade‐off as many species have large foraging ranges and their prey often have a patchy distribution. This environment leads these species to exhibit pronounced exploration and exploitation phases but differences between individuals are poorly resolved. Personality differences are known to be important in foraging behaviour but also in the trade‐off between exploration and exploitation. Here we test whether personality predicts an individual exploration–exploitation strategy using wide ranging wandering albatrosses (*Diomedea exulans*) as a model system.
3. Using GPS tracking data from 276 wandering albatrosses, we extract foraging parameters indicative of exploration (searching) and exploitation (foraging) and show that foraging effort, time in patch and size of patch are strongly correlated, demonstrating these are indicative of an exploration–exploitation (EE) strategy. Furthermore, we show these are consistent within individuals and appear stable in the population, with no reproductive advantage.
4. The searching and foraging behaviour of bolder birds placed them towards the exploration end of the trade‐off, whereas shy birds showed greater exploitation. This result provides a mechanism through which individual foraging strategies may emerge. Age and sex affected components of the trade‐off, but not the trade‐off itself, suggesting these factors may drive behavioural compensation to maintain resource acquisition and this was supported by the evidence that there were no fitness consequence of any EE trait nor the trade‐off itself.
5. These results demonstrate a clear trade‐off between information gathering and exploitation of prey patches, and reveals for the first time that boldness may drive these differences. This provides a mechanism through which widely reported links between personality and foraging may emerge.

## 1 INTRODUCTION

Finding food is essential in most species for reproduction and survival. In nature, prey is often distributed in discrete patches and the ability to efficiently exploit such resources will be under natural selection (Charnov, 1976; Krebs, 1978). Classic optimal foraging theory predicts that the way in which animals allocate their time within and between patches will be dependent on the quality of a patch and the distribution of patches in the environment (Marginal Value Theorum; Charnov, 1976). However, individuals do not have complete knowledge about the environment and so they must gather such information constantly (Dall, Giraldeau, Olsson, McNamara, & Stephens, 2005; Krebs, Kacelnik, & Taylor, 1978; Lima, 1984). This results in a trade‐off between obtaining information about where to feed (exploration) and feeding itself (exploitation : The exploration–exploitation (EE) trade‐off; Cohen, McClure, & Yu, 2007; Eliassen, Jørgensen, Mangel, & Giske, 2007; Kramer & Weary, 1991; Mehlhorn et al., 2015).

Research across the animal kingdom has shown that the three main drivers which influence exploration and exploitation are the nature of the environment, social factors and individual differences (Mehlhorn et al., 2015). Individual level drivers in animals include morphology (Armstrong, Braithwaite, & Huntingford, 1997; Riveros & Gronenberg, 2010), state or motivation (Bacon, Hurly, & Healy, 2010; Caraco, 1981; Caraco et al., 1990), cognitive ability and memory (Hills & Pachur, 2012; Rakow, Newell, & Zougkou, 2010) and neurotransmitters (Hills, 2006). While consistent individual differences in behaviour, or personality, often capture components of exploration and exploitation, they have not been directly linked to this trade‐off.

Exploration, as part of the EE trade‐off, captures suites of movement traits between foraging patches (Mehlhorn et al., 2015). Measurement of exploration as a personality trait itself is most commonly conducted in an open field test, when exploration of a novel environment is captured (Carter, Feeney, Marshall, Cowlishaw, & Heinsohn, 2013; Verbeek, Drent, & Wiepkema, 1994). These tests are carried out in standardised conditions in an attempt to control for environmental variation. In many species, where foraging behaviour itself can be readily measured, testing exploration of a novel environment is challenging. While differences in foraging trip duration and distance travelled (Patrick & Weimerskirch, 2014) could be interpreted as exploration in a known environment, these are strongly confounded by other variables and hence conclusions in relation to personality traits should be treated with caution. Personality can also be measured using different assays and these can be grouped in the correlated (syndromes; Sih, Bell, Johnson, & Ziemba, 2004) or uncorrelated (Carter et al., 2013) aspects. Behavioural syndromes offer an opportunity to assay traits thought to be linked to exploration of a novel environment indirectly through correlated traits.

Since early studies highlighting the existence of behavioural syndromes, evidence has been building, both supporting (e.g. Class & Brommer, 2015; Dochtermann & Jenkins, 2007) and questioning the generalisation of these suites of traits (Carter et al., 2013). One of the drivers of this conflict is the failure to adequately define personality traits. Boldness in particular has a multitude of meanings, most notably being used to describe both the response to a novel object (sensu neophobia) and risk taking and anti‐predator response (Carter et al., 2013). Measurements of boldness in response to a novel object or using a neutral human approacher as a novel object are available on wide ranging species (Patrick, Charmantier, & Weimerskirch, 2013; Patrick & Weimerskirch, 2014). Although the response to a novel object is often correlated with exploration of a novel environment, it can be independent of risk taking/anti‐predator behaviour (Carter et al., 2013). However, we hypothesis that boldness in response to a novel object (hereafter “boldness”) will correlate with the EE trade‐off, if this boldness correlates with exploration in a novel environment and hence exploration in natural systems.

Both boldness and exploration of a novel environment have been shown to correlate with different components with both large scale movement strategies such as dispersal (Cote, Clobert, Brodin, Fogarty, & Sih, 2010; Cote, Fogarty, Tymen, Sih, & Brodin, 2013; Dingemanse, Both, van Noordwijk, Rutten, & Drent, 2003; Fraser, Gilliam, Daley, Le, & Skalski, 2001; Quinn, Cole, Patrick, & Sheldon, 2011), or migratory behaviour (Chapman et al., 2011), fine scale movement such as space and habitat use (Patrick & Weimerskirch, 2014; Spiegel, Leu, Sih, Godfrey, & Bull, 2015) and individual (Dammhahn & Almeling, 2012; Patrick & Weimerskirch, 2014, 2015) and group foraging (Aplin, Farine, Mann, & Sheldon, 2014; Kurvers et al., 2010, 2010). In general these results show that bolder individuals have a greater propensity for wide scale movement, suggesting a link to the exploration aspect of the EE trade‐off (Mazué, Dechaume‐Moncharmont, & Godin, 2015; Sih et al., 2004; Verbeek et al., 1994; Wilson & Godin, 2009; but see Carter et al., 2013).

Evidence of boldness and exploration of a novel environment linking to an EE trade‐off can also be found indirectly, through the association between boldness and risk taking (Dammhahn & Almeling, 2012; Reale, Reader, Sol, McDougall, & Dingemanse, 2007; Sih et al., 2004; Sloan Wilson, Clark, Coleman, & Dearstyne, 1994; Wilson et al., 2010). While this correlation between boldness and risk‐taking is not always present (Carter et al., 2013) individuals favouring exploration will gain incomplete information from the environment, due to rapid, superficial assessment, such that making decisions based on this is risky. This can be applied specifically to foraging in a heterogeneous landscape as prior foraging success may be a relatively reliable indicator of future success, but as the returns will diminish with consumption, a riskier strategy would be to move frequently between patches, with a high‐risk–high‐gain tactic indicative of exploration (Mehlhorn et al., 2015; Tuttle, Wulfson, & Caraco, 1990).

Of the studies which have examined the link between these personality traits and exploitation, the strength of the relationship is mixed, and often varies in direction depending on the environment. For example, in birds, fast exploring individuals have been reported to remain at a single food source longer than slow explorers and take longer to discover new prey patches, when food is plentiful, suggesting high exploitation propensities (Drent & Marchetti, 1999; Herborn et al., 2010; Marchetti & Drent, 2000; Verbeek et al., 1994). However, evidence also shows that when these food sources are removed, mimicking food depletion, it is the slow explorers who repeatedly revisit these sites (van Overveld & Matthysen, 2013) and that fast birds switch foraging location more rapidly, travelling further to find new foraging areas (van Overveld & Matthysen, 2010). This can be used as indirect evidence that bolder individuals would favour the strategy of fast explorers under these scenarios, whereas shyer individuals continue to exploit patches, despite reduction in prey availability, akin to slower explorers. In nature, food patches are predicted to deplete, and movement between patches would only be under selection if this is the case (Charnov, 1976). Therefore, we suggest that these studies, which mimic natural food depletion, have the potential to capture natural variation between personality types, resulting in the hypothesis that boldness will correlate negatively with exploitation.

**Figure 1:**
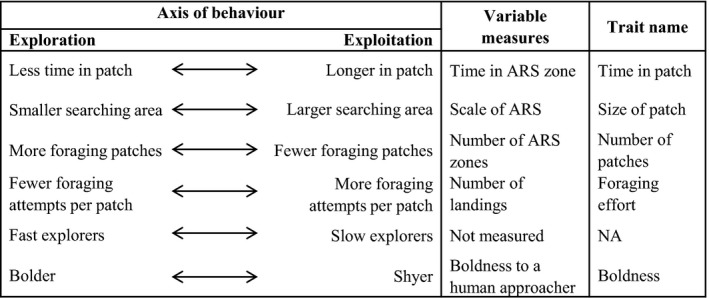
The potential behavioural strategy linking boldness traits with exploration and exploitation of foraging patches. ARS = area‐restricted search

However, despite the large body of evidence in the personality literature, suggesting that heritable behavioural differences could result in individuals who differ in aspects of the EE trade‐off, there have been few attempts to directly link it to personality. For personality differences to persist individuals should have equal fitness at equilibrium (Dingemanse & Reale, 2005; Wolf, van Doorn, Leimar, & Weissing, 2007), leading to the prediction that aspects of foraging strategies will fall along this trade‐off. Furthermore, evidence that personality types are favoured under different environments leads to predictions that the exploitation of resources that vary in time and space may favour individuals at different ends of the EE trade‐off. As environment will interact with foraging behaviour, a proportion of this trade‐off may be mediated by habitat and social effects, yet we predict that inherited personality differences will drive consistent differences in searching and foraging, and hence the EE trade‐off. In Figure 1 we outline the predictions, based on the current literature, creating a testable framework of how suites of foraging traits may vary as a function of personality and an individual's place along the EE trade‐off. To fully understand whether the EE trade‐off is mediated by personality, aspects of both exploration and exploitation of resources must be simultaneously measured, alongside assays of personality. Furthermore, to assess the fitness consequences of this trade‐off, measures of reproductive success are required. Seabirds are an ideal species for a study to examine the movement between foraging patches as they can cover over 8 million km in their lifetime (Weimerskirch et al., 2014) and their movement can be readily captured using GPS loggers (Hart & Hyrenbach, 2010; Rutz & Hays, 2009). In the marine environment, prey have a predictable yet patchy distribution, leading to an environment where patch switching is adaptive (Weimerskirch, 2007).

In this study, we assess the presence of an EE trade‐off in wandering albatross (*Diomedea exulans*) examining components of the trade‐off and associations between traits. We assess whether these represent alternative stable strategies, linking them to fitness and foraging trip metrics, exploring whether boldness, age and sex can account for individual differences. We predict that birds that favour exploration will spend less time in each foraging patch, use more numerous but smaller patches, with a lower foraging effort (landings per foraging patch). Bolder birds will fall at the exploration end of the trade‐off with shyer birds at the exploitation end. We predict these will represent searching strategies, but not foraging success nor resource acquisition and will therefore show no correlation with fitness.

## 2 MATERIALS AND METHODS

### 2.1 Study site and species

The study was conducted on a population of wandering albatrosses, a large long lived seabird (8–12 kg), on Possession Island, Crozet Archipelago (46°S, 51°E) between 2008 and 2016. Here ca. 350 wandering albatross pairs breed every year. Since the species is a biennial breeder, that is, it breeds every second year when it successfully raises a chick, this results in a total breeding population (across 2 years) of *c*. 1,200 individuals. All birds are individually marked and pair ID and reproductive success is recorded annually. This species is mainly a solitary feeder (Weimerskirch, Jouventin, & Stahl, 1986). Wandering albatross foraging trips are comprised of long straight movements at high speed, interspersed with periods of intensive searching (Pinaud & Weimerskirch, 2007; Weimerskirch, Pinaud, Pawlowski, & Bost, 2007), indicative of a suite of EE traits, and such foraging behaviours have been suggested to be part of an EE trade‐off (Mehlhorn et al., 2015).

### 2.2 Foraging behaviour

From 2010 to 2016, 276 adult wandering albatrosses were equipped with GPS loggers (2010—9 birds; 2011—39 birds; 2012—25 birds; 2013—109 birds; 2014—19 birds; 2015—16 birds; 2016—59 birds; IgotU 120/600, Mobile Action Technology), during incubation. Loggers were waterproofed in heat shrink tubing and attached using Tesa tape to the lower back. Devices recorded a location every 15 min for an entire foraging trip. GPS loggers were mainly left on birds for a single foraging trip and on return, birds were recaught and the devices retrieved and downloaded. All points recorded on land (at the nest) were removed. The mean trip duration was 10.98 ± 6.99 days. Trip duration was defined at the time a bird was away from the colony for a single trip. Total distance was the total distance travelled, calculated by summing the distance between successive GPS points. Maximum range was the maximum distance from the colony to any GPS location.

The most comprehensive literature considering the EE trade‐off comes from studies examining area‐restricted search (Mehlhorn et al., 2015). Area‐restricted search captures changes from extensive searching (exploration) to intensive searching (exploitation) and has been used widely to quantify foraging and searching behaviour in albatrosses (Pinaud & Weimerskirch, 2005, 2007; Weimerskirch et al., 2007). The scale and location of the areas can be identified by peaks in first passage time. First passage time is the time taken to travel across a circle of given radius, and peaks in first passage time show changes from straight to tortuous movement. The scale at which searching is most intense can be identified by calculating the first passage time at each data point, across a circle of varying radius (Fauchald & Tveraa, 2003).

Briefly, first passage time must be calculated on trajectories where points are equidistant apart and here we interpolated to a distance of 1 km. We also removed all time periods where the birds were on the water (speed filters <10 km/hr) as increased turning rate during these periods is usually indicative of local movement at the surface due to ocean currents, not active searching. We examined circles ranging from 2 to 400 km and plotted the variance in log (first passage time) against the circle radius to estimate the scale at which the maximum variance in log (first passage time) occurs. From this, the scale of area‐restricted search (scale at which peak variance (log [first passage time] occurs) was estimated and this was used as the “*size of patch*.” Each track was divided into homogeneous segments (in terms of mean and variance in first passage time) using the Lavielle segmentation (Barraquand & Benhamou, 2008) implemented in the R package adehabitatLT (Calenge, 2006). These segments were then considered to be periods of area‐restricted search if the mean first passage time during this period was higher than the mean across the foraging trip. Periods where the first passage time was lower than the mean were considered to be non‐area‐restricted search zones (Pinaud & Weimerskirch, 2005, 2007; Weimerskirch et al., 2007). In zones of area‐restricted search we calculated the number of landings (Speed <10 km/h) per zone—a proxy of energetic costs (Shaffer, Costa, & Weimerskirch, 2003), and this was used as “foraging effort.” The time spent in area‐restricted search zones was measured as the time the bird exited the area‐restricted search zone—the time the bird entered the zone and this was used as “*time in patch*.” The number of area‐restricted search zones per trip was used as an estimate of the “*number of patches*” (Figure 1).

In total 816 foraging patches were identified from 276 individual trips, with a mean of 3.15 ± 2.10 patches per individual per trip. We subset the data to include measures of EE trade‐off components per foraging trip, with a measure of number of patches and size of patch calculated across the whole trip, and randomly selected measures of time in patch and foraging effort. We also conducted the analyses on the full dataset and a dataset subsampled to include only one randomly selected trip per bird (N patches = 292; N trips = 274; N birds = 228). These results supported those in the main paper (Appendix 1 Table S1; Appendix 2 Tables S1–S6) and full details of sample sizes for each analysis and given in all table legends.

### 2.3 Boldness

From 2008 to 2016 an individual's place along the shy‐bold continuum was measured as the level of responsiveness and aggression towards a neutral human approacher. Bold birds were highly responsive and shy birds showed little response (Patrick et al., 2013). The behavioural response was scored along an ordinal scale from 0—no response, 1—lifts head, 2—stands on tarsus, 3—vocalises, 4—stands up. The presence of any of these behaviours was recorded to produce a series of scores per individual and the maximum score was used as an estimate of boldness. We demonstrated that the score represents a progressive increase in responsiveness, as sequential behaviours were generally observed. For example, an individual which lifted its head (1) and vocalised (3), generally also raised up on its tarsus (2). All tests were conducted while birds were incubating to avoid the response of the chick confounding adult behaviour. The number of tests per bird was not controlled as a test was conducted on all birds present at each of the three annual demographic controls and on deployment and recovery of GPS devices. We used the response to a human approacher as it allowed large numbers of birds to be tested per season. In 2013 we also conducted a novel object test, using a large inflatable blue cow. This test measured the response to a novel object with a human 3 m behind and lying flat on the ground. In seabirds it is very difficult to use a novel object approach without a human present. These results showed a strong correlation with the response to a human but birds were on average more aggressive to the novel object (S. Patrick, unpbl. data). Birds assigned as bold from the human approach test showed pronounced aggression to the novel object, whereas shy birds showed little response, providing strong support that our measure of boldness persisted across contexts. In total 1,113 individuals were tested over a series of 3,777 tests, generating robust evidence of differences in boldness. Observer and observation number have previously been shown to have significant effects of boldness and were hence fitted in all models (Patrick et al., 2013). This score has previously been shown to be repeatable (*R*: 0.45 [CI: 0.38–0.51]) and heritable (*h* ^2^ = 0.24 [CI: 0.05–0.41]) across a large number of individuals (See Patrick et al., 2013 for further details). We estimated individual measures of boldness using a GLMM, with observer, observation number and bird ID as fixed estimates and extracted individual parameter estimates which were mean centred at the population level (1,113 individuals) to produce boldness estimates (See Patrick et al., 2013 for further details). These were used in preference to Best Unbiased Linear Predictors as these have been shown to be unsuitable estimates (Hadfield, Wilson, Garant, Sheldon, & Kruuk, 2010).

### 2.4 Statistics

All explanatory and response variables were standardised to have a mean of 0 and a standard deviation of 1. *p* values are provided for all analyses. Estimates for log transformed data are given on the logged scale. In GLMMs the significance of all effects was calculated using ANOVA comparisons between models with and without the term of interest. All first order interactions were dropped when non‐significant.

#### 2.4.1 EE trade‐off and individual strategy

We estimated the correlation between four foraging traits (size of patch, time in patch, number of patches and foraging effort) using Pearson's correlation coefficients and associated *p* values to examine whether there was evidence of correlated traits within the trade‐off. A positive correlation is representative of groups of traits being consistently displayed by the same individuals. We also used the R package prcomp to conduct a principal component analysis to extract a single metric representative of an EE strategy. Models were also run as multivariate analyses in MCMCglmm (Hadfield, 2010), and the covariation between foraging traits and personality estimated. Using multivariate models we failed to reach full convergence, however, results supported those presented here (Appendix 3; Table S1). We assessed the repeatability of all individual trade‐off components and individual EE strategies using the package rptR (Nakagawa & Schielzeth, 2010). Models included boldness, age and sex, and where appropriate the interaction between age and sex (See below for full details). We used bootstrapping without randomisation (1,000 iterations) to estimate confidence intervals for repeatability estimates and used likelihood ratio tests to calculate *p* values.

#### 2.4.2 Drivers of EE trade‐off and individual strategy

Boldness, age, sex and the interaction between age and sex (known to have a significant effect on foraging behaviour in this species (Weimerskirch et al., 2014) were all fitted, where possible, in GLMMs, using lme4 (Bates & Maechler, 2010) and nlme (Pinheiro, Bates, DebRoy, & Sarkar, 2015), with bird ID and year as random effects and as response variables: (1) EE strategy (PC1; log[*x* + 2] transformed), (2) time in patch (3) foraging effort (4) size of patch and (5) number of patches.

Trip duration (days), total distance travelled (km) and maximum range (maximum distance from the colony; km) were all fitted individually in GLMMs (due to strong covariation between these metrics) with EE strategy (PC1; log[*x* + 2] transformed) as the response. Bird ID and year were included as random effects.

#### 2.4.3 Fitness consequences of EE trade‐off and individual strategy

Fitness consequences were modelled with reproductive success in the year of tracking (binary) fitted as the response in a binary GLMM. EE strategy (PC1; log[*x* + 2] transformed), time in patch, number of patches, foraging effort and size of patch as fixed effects in individual models (traits covaried). Bird ID and year were included as random effects but we did not include any fixed effects in our models as there is no evidence of linear effects of sex or age on reproductive success in this population. Partner ID was not included as a random effect as the extremely high levels of monogamy mean this does not change over the time frame used in the study.

## 3 RESULTS

### 3.1 EE trade‐off

We found strong support for the hypothesis that albatrosses display a detectable EE trade‐off. Individuals which spent longer in a patch had larger patches (*r* = .37; *p* < .001) and had a higher foraging effort per patch (*r* = .57; *p* < .001; Table 1). There was also a positive correlation between size of patch and foraging effort (*r* = .33; *p* < .001; Table 1). However, there was no correlation between any of these traits and the number of patches (*r* = −.06–.01; *p* = .34–.86; Table 1). A principal component analysis showed evidence of a strategy where the time in patch (0.62), foraging effort (0.60) and size of patch (0.50) were positively weighted, with little effect of the number of patches (−0.05; Table 2). This principal component explained almost half the variation in the EE trade‐off (0.47; Table 2) and this was used as a measure of the EE strategy, indicative of the level of exploitation (negative = exploration; positive = exploitation).

**Table 1:** The correlations among foraging traits for all individuals in the population. Pearson's correlation coefficients are shown on the top half of the matrix and *p* values on the bottom half. Dataset includes one measure of EE traits per trip (N patches = 292; N trips = 274; N birds = 228). Significant values are shown in bold

Foraging variable	Time in patch	Foraging effort	Size of patch	Number of patches
Time in patch		**0.57**	**0.37**	‐0.06
Foraging effort	**<0.001**		**0.33**	‐0.03
Size of patch	**<0.001**	**<0.001**		0.01
Number of patches	0.34	0.60	0.86	

**Table 2:** Principal components (PC), weightings and variance explained from a principal components analysis. PC1 is used in the paper as a proxy for EE strategy. Dataset includes one measure of EE traits per trip (N patches = 292; N trips = 274; N birds = 228)

Foraging variable	PC1	PC2	PC3	PC4
Time in patch	0.62	−0.03	0.29	−0.72
Foraging effort	0.60	0.01	0.41	0.68
Size of patch	0.50	0.14	−0.85	0.07
Number of patches	−0.05	0.99	0.13	0.04
Proportion of variance explained	0.47	0.26	0.16	0.12
Cumulative variance explained	0.47	0.72	0.88	1.00

We found that all foraging traits and a bird's EE strategy itself were repeatable within individuals. Size of patch (*R* = 0.71 (0.57–0.82); *p* < .001; Table 3), number of patches (*R* = 0.48 (0.31–0.66); *p* < .001; Table 3) and EE strategy (*R* = 0.40 (0.27–0.54); *p* < .001; Table 3) were most repeatable, with time in patch (*R* = 0.25 (0.15–0.37); *p* < .001; Table 3) and foraging effort (*R* = 0.21 (0.12–0.30); *p* < .001; Table 3) showing significant but lower repeatabilities.

**Table 3:** Variance components (±*SE*) and repeatability (Confidence intervals) extracted from final models for foraging traits, boldness, sex and age (See Table 4). N patches = 197; N trips = 186; N birds = 159

	Random effect variance estimates			Repeatability
Response variable	Bird ID	Year	Residual	Bird ID R
PC1 (EE strategy)	0.16 ± 0.40	0.03 ± 0.19	0.19 ± 0.44	0.40 (0.27, 0.54); *p* < .001
Time in patch	0.30 ± 0.55	0.05 ± 0.22	0.42 ± 0.65	0.25 (0.15, 0.37); *p* < .001
Foraging effort	0.45 ± 0.67	0.00 ± 0.00	0.69 ± 0.83	0.21 (0.12, 0.30); *p* < .001
Size of patch	0.32 ± 0.56	0.06 ± 0.24	0.23 ± 0.48	0.71 (0.57 0.82); *p* < .001
Number of patches	0.05 ± 0.21	0.03 ± 0.16	0.14 ± 0.38	0.48 (0.31, 0.66); *p* < .001

### 3.2 Intrinsic drivers of the EE trade‐off

**Figure 2:**
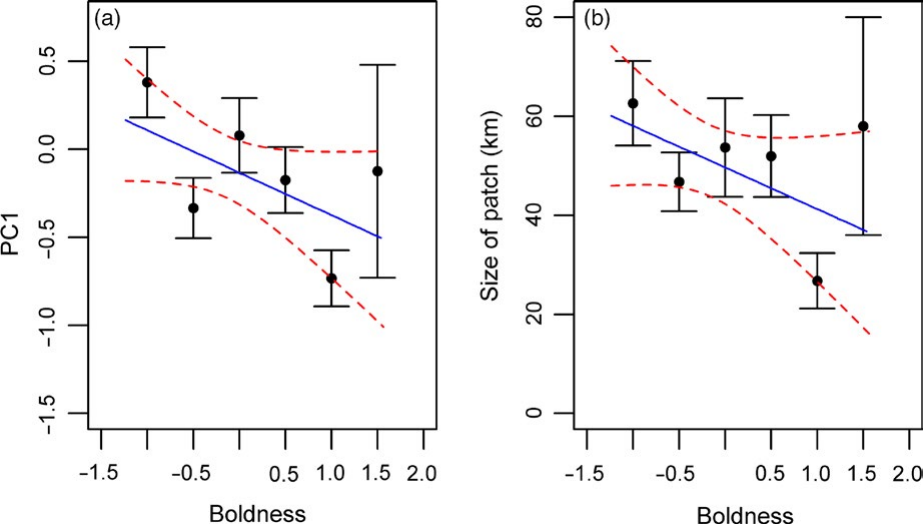
Boldness and foraging traits: (a) Boldness and principal component one, indicative of an individual's position along an exploration–exploitation trade‐off (*p* = .045). (b) The size of foraging patches in relation to individual boldness (*p* = .026). For plotting purposes only, boldness scores are grouped and shown as the mean with standard errors (Boldness groups [N]: [−1.5, −1.0] (42); [−1.0, −0.5] (56); [−0.5, 0.0] (43); [0.0, 0.5] (35); [0.5, 1.0] (18); [1.0, 1.5] (3)). Raw data are shown in Appendix 2 Figure S1. The model prediction is plotted as a line with dashed 95% confidence intervals of the predicted line [Colour figure can be viewed at wileyonlinelibrary.com]

Boldness correlated with EE strategy, indicative of differences in the position of these individuals along the EE trade‐off (χ_1_^2^ = 4.01; *p* = .045; Table 4) with shyer individuals having higher values (Estimate: −0.14 ± 0.07; Table 4; Figure 2a), demonstrating a lower foraging effort, less time in each patch and smaller patches (i.e. exploitation). There was no effect of age (χ_1_^2^ = 0.76; *p* = .38; Table 3) or sex (χ_1_^2^ = 0.41; *p* = .52; Table 4) nor the interaction between these χ_1_^2^ = 1.77; *p* = .18; Table 4) on EE strategy.

**Table 4:** The relationship between boldness, age and sex with component traits of the EE trade‐off and principal component one (EE strategy). Estimates from general linear mixed models of slopes and intercepts are presented for all effects and significant results are shown in bold. Non‐significant interactions were dropped from all models and are shown in italics. Dataset includes one measure of EE traits per trip (N patches = 197; N trips = 186; N birds = 159). M = male; F = female

		Explanatory variables			
Response variable	Model output	Boldness	Age	Sex	Age x Sex
PC1 (EE strategy)	Test statistic	**χ** ^**2**^_**1**_ ** = 4.01;**	χ^2^_1_ = 0.76;	χ^2^_1_ = 0.41;	χ^2^_1_ * =* 1.77;
	*p* value	***p*** ** = .045**	*p* = .38	*p* = .52	*p = *.18
	Estimate ± *SE* (logged scale)	**−0.09 ± 0.05**	0.05 ± 0.05	F:0.48 ± 0.10 M:0.54 ± 0.10	−0.01 ± 0.01
Time in patch	Test statistic	χ^2^_1_ = 2.58;			**χ** ^**2**^_**1**_ **= 4.59;**
	*p* value	*p* = .11			***p*** ** = .032**
	Estimate ± *SE* (logged scale)	−0.10 ± 0.07			**−0.34 ± 0.16**
Foraging effort	Test statistic	χ^2^_1_ = 2.84;	χ^2^_1_ = 0.15;	χ^2^_1_ = 0.44;	χ^2^_1_ = 0.12;
	*p* value	*p* = .09	*p* = .71	*p* = .51	*p = *.73
	Estimate ± *SE* (logged scale)	−0.14 ± 0.08	−0.04 ± 0.09	F:‐0.50 ± 0.11 M:‐0.61 ± 0.12	−0.07 ± 0.20
Size of patch	Test statistic	**χ** ^**2**^_**1**_ **= 4.98;**	χ^2^_1_ = 1.20;	χ^2^_1_ = 2.32;	χ^2^_1_ * = *0.90*;*
	*p* value	***p*** ** = .026;**	*p* = .27	*p* = .13	*p* = .34
	Estimate ± *SE* (logged scale)	**−0.13 ± 0.06**	0.07 ± 0.07	F:‐0.42 ± 0.13 M:‐0.24 ± 0.13	−0.13 ± 0.14
Number of patches	Test statistic	χ^2^_1_ = 0.11;	χ^2^_1_ = 1.41;	**χ** ^**2**^ ** = 4.23;**	χ^*2* ^= 0.01;
	*p* value	*p* = .74	*p* = .24	***p*** ** = .040**	*p = *.93
	Estimate ± *SE* (logged scale)	0.01 ± 0.03	−0.04 ± 0.00	**F:0.57 ± 0.08** **M:0.43 ± 0.08**	0.01 ± 0.08

**Figure 3:**
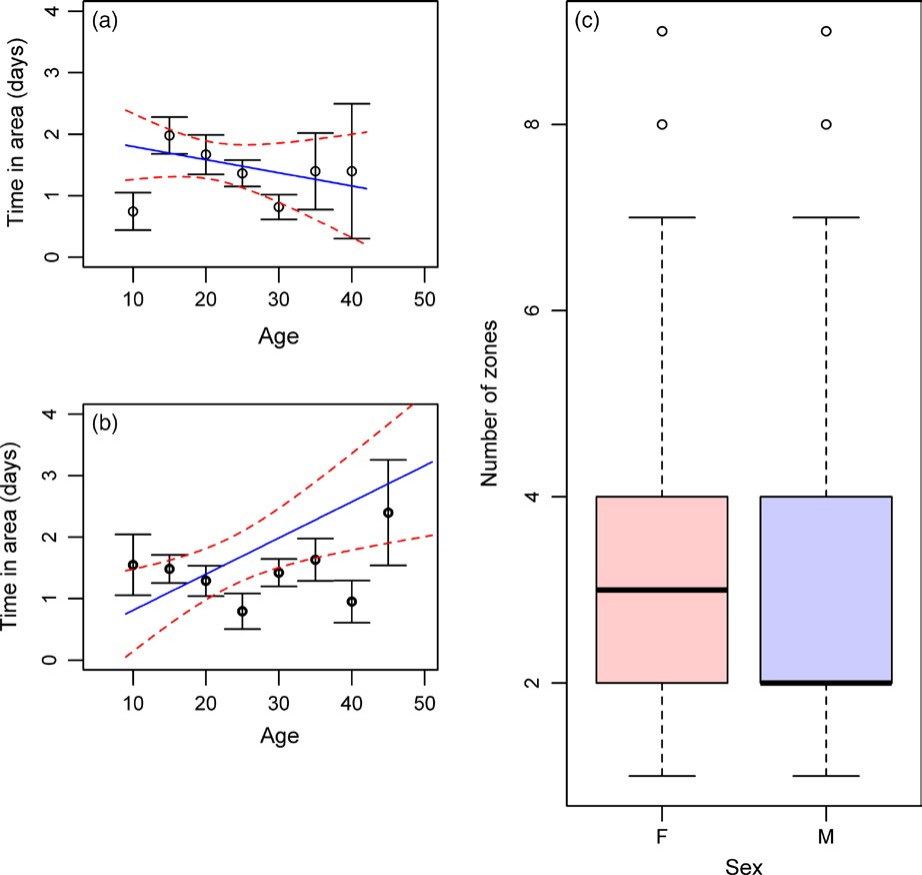
Age and sex effects on foraging traits. (a) Males show a weak decrease in time in patch with increasing age. N: [0–10 years] (6), [10,15] (33), [15, 20] (25), [20, 25] (9), [25, 30] (10), [30, 35] (7), [35, 40] (3). (b) Females show a strong increase in time in patch as they age. N: [0–10 years] (13), [10, 15] (23), [15, 20] (22), [20, 25] (9), [25, 30] (16), [30, 35] (13), [35, 40] (3), [40 45] (4) (*p* = .032). There is an outlying value for a female of 51 years which is not displayed (time in patch = 19.4 days). (c) Females have more foraging zones per trip than males (*p* = .040) [Colour figure can be viewed at wileyonlinelibrary.com]

Examining each trait individually, boldness correlated with the size of foraging patches (χ_1_^2^ = 4.98; *p* = .026; Estimate = −0.13 ± 0.03; Figure 2b; Table 4), with bolder birds having smaller foraging patches. There was no relationship with time in patch (χ_1_^2^ = 2.58; *p* = .11; Table 4), foraging effort (χ_1_^2^ = 2.84; *p* = .09; Table 4) nor the number of patches (χ_1_^2^ = 0.11; *p* = .74; Table 4). There was an interaction between age and sex on the time in patch with a weak effect that older males spent less time in a patch (χ_1_^2^ = 4.59; *p* = .032; Table 4; Figure 3a) but females spent longer in patches as they age (Figure 3b). Males also had fewer patches than females (χ_1_^2^ = 2.58; *p* = .11; Table 4; Figure 3c). There were no age or sex effects on any other trait (Table 4).

### 3.3 Foraging behaviour, fitness and the EE trade‐off

**Figure 4:**
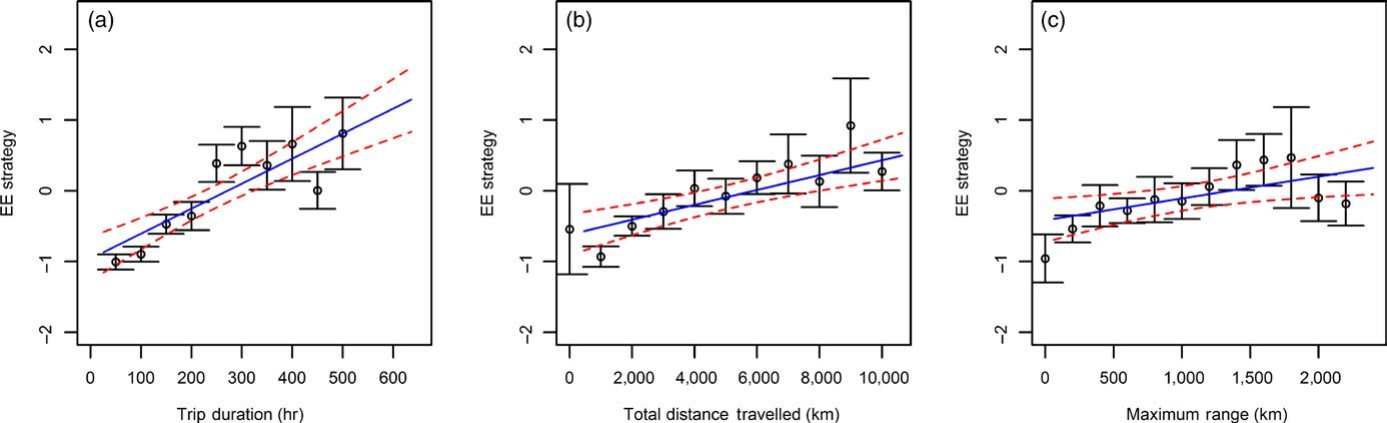
The relationship between EE strategy and foraging trip matrices. (a) Individuals which favour exploitation have longer foraging trip durations (*p* < .001). N: [0, 50] (0), [50, 100] (11), [100, 150] (35), [150, 200] (28), [200, 250] (28), [250, 300] (31), [300, 350] (20), [350, 400] (15), [400, 450] (9), [450–840] (7), [500–840](13). (b) Individuals which favour exploitation travel further during trips (*p* < .001). N: [0,1000] (3), [1000, 2000] (21), [2000, 3000] (33), [3000, 4000] (24), [4000, 5000] (26), [5000, 6000] (19), [6000, 7000] (14), [7000, 8000] (15), [8000, 9000] (9), [9000, 10000] (12), [10000–17700] (21). (c) Individuals which favour exploitation have larger maximum ranges (*p* < .001). N: [0, 200] (6), [200, 400] (31), [400, 600] (17), [600, 800] (24), [800, 1000] (15), [1000, 1200] (16), [1200, 1400] (18), [1400, 1600] (17), [1600, 1800] (23), [1800, 2000] (8), [2000, 2200] (11), [2200–3940] (11). Foraging metrics are grouped for plotting purposes. Raw data are shown in Appendix 2 Figure S2 [Colour figure can be viewed at wileyonlinelibrary.com]

There was a strong positive correlation between EE strategy and trip duration (χ^2^_1_ = 33.25; *p* < .001), total distance travel per trip (χ^2^_1_ = 24.41; *p* < .001) and the maximum range (χ^2^_1_ = 14.82; *p* = .001). This showed that individuals favouring exploitation had longer trip duration (Estimate [logged scale] ± *SE*: 0.23 ± 0.04; Figure 4a), travelled further in total (Estimate [logged scale] ± *SE*: 0.19 ± 0.04; Figure 4b) and had a larger maximum range (Estimate [logged scale] ± *SE*: 0.14 ± 0.04; Figure 4c).

There was no evidence of reproductive correlates of the EE strategy (PC1; χ^2^_1_ = 0.05; *p* = .82; Table 5), nor any of the components individually (time in patch: χ^2^_1_ = 0.00; *p* = .95; foraging effort: χ^2^_1_ = 2.33; *p* = .13; size of patch: χ^2^_1_ = 0.64; *p* = .42; number of patches: χ^2^_1_ = 0.32; *p* = .57; Table 5).

**Table 5:** The relationship between the EE strategy and individual components with reproductive success. Estimates from general linear mixed models of slopes and intercepts are presented for all effects and significant results are shown in bold. Dataset includes one measure of EE traits per trip (N patches = 282; N trips = 267; N birds = 223)

Response variable	Explanatory variables	Test statistic (*df*)	*p* value	Estimate ± *SE* (logged)
Reproductive success	PC1 (EE strategy)	χ^2^_1_ = 0.05	.82	‐0.09 ± 0.40
	Time in patch	χ^2^_1_ = 0.00	.95	0.01 ± 0.18
	Foraging effort	χ^2^_1_ = 2.33	.13	‐0.26 ± 0.17
	Size of patch	χ^2^_1_ = 0.64	.42	0.14 ± 0.19
	Number of patches	χ^2^_1_ = 0.32	.57	0.10 ± 0.18

## 4 DISCUSSION

Our results provide comprehensive evidence that albatrosses show an EE strategy, with the size of patches, the time in patches and foraging effort all covarying with one another, but no correlation with the number of patches. We show that bolder wandering albatrosses have smaller foraging patches, which is in keeping with our predictions that they would tend to show greater exploration and this was confirmed by the association between boldness and the general EE strategy. High exploration, shown by birds with low EE strategy values, was found in birds with shorter trips in terms of time and distance, and exploitation was associated with longer trips. Interestingly, while boldness did not predict the time in patch and number of patches, these were instead explained by age and sex differences. Despite this there was no association between reproductive success and EE strategy or any of its individual components. These results show a clear EE strategy in albatrosses and previous studies showing these trait correlations represent a trade‐off suggests that exploration and exploitation are a foraging trade‐off in this species. Together these results demonstrate that boldness is a strong predictor of EE strategy and suggesting adaptive age and sex differences in components of this trade‐off.

### 4.1 Boldness and EE strategies

The results showing that boldness correlates with traits in the EE trade‐off and with a general EE strategy strongly support the hypothesis that bolder birds lie at the exploration end of the trade‐off, confirming our predictions. These data support previous results showing that bolder individuals explore relatively superficially (Mazué et al., 2015; Reale et al., 2010; Verbeek et al., 1994) and that boldness correlates with exploration in a novel environment (Sih et al., 2004; Verbeek et al., 1994). As bolder animals have been shown to be more risk taking (Dammhahn & Almeling, 2012; Sih et al., 2004) this may drive their tendency to favour exploration as continually moving between patches may also be a risky strategy, particularly when prey have a patchy distribution, as new foraging patches bring unknown reward. Previous results have also suggested that fast exploring individuals discover new patches quicker when food is limited (van Overveld & Matthysen, 2010, 2013). This result suggests that given correlation between boldness and exploration, these results may be mirrored in bold birds in the natural environment, where prey depletion is common.

### 4.2 Boldness and the EE trade‐off

While there are many studies which examine individual differences in single components of an EE trade‐off (Reviewed by Mehlhorn et al., 2015), studies testing differences in EE strategies between individuals are rarer. Examining single foraging traits can bias our understanding of the adaptive consequences of individual differences as they ignore any trade‐off with associated traits. Studying foraging effort per patch without simultaneously measuring the size of patch or time in patch, may erroneously imply differences in foraging investment. In this study we show that EE strategy and all component traits are repeatable within individuals, and yet the number of patches is not part of the EE trade‐off. This provides strong support that an individual's place along the trade‐off is stable over time. However, given individuals also show a repeatable number of patches we suggest this trait may be explained by other variables such individual foraging habitat or efficiency associated with age or sex.

### 4.3 Age and sex drivers of the EE trade‐off

The EE trade‐off has been identified in other species (Reviewed by Mehlhorn et al., 2015) and individual differences have been proposed to occur as a result of factors such as cognitive capacity, aspiration, physiology, morphology and age (Reviewed by Mehlhorn et al., 2015). Foraging strategies in wandering albatrosses are known to vary with both age and sex (Lecomte et al., 2010; Patrick & Weimerskirch, 2015; Weimerskirch et al., 2014) and here we find components of EE strategies are linked to age and sex, but not the trade‐off itself. Previous work have shown bolder birds increase the duration of foraging trips as they age (Patrick & Weimerskirch, 2015), and this is postulated to be adaptive for males as they travel further south, supported by evidence these bolder males show less pronounced senescence, unlike older females who visit the less productive tropics. Our results show females spend longer in patches as they age, whereas males show a weak decrease in time as they get older. This may be evidence of a need for females to increase effort as a result of poorer broad quality habitat, and this is supported by our results showing females have more patches than males. It is only these two components of the trade‐off, not the trade‐off itself that correlates with age and sex, which may show a decoupling of the trade‐off when an individual's ability to acquire sufficient resources alter foraging behaviour. As the sexes exploit different habitats and hence have the potential for different prey distributions, these may drive changes in the traits most closely linked to energy gain. For adaptive consequences of these differences to be identified, the emergence of senescence in conjunction with these differences in EE strategies would be an exciting test of the causes and consequence of this variation.

### 4.4 The EE trade‐off and foraging behaviour

We predicted that EE strategies, which represent the way in which birds search for food, gather information and exploit detected patches would not correlate with foraging trip length. However, our results show that explorative individuals have shorter foraging trips in both distance and duration. The cause and effect of such a relationship is difficult to decouple. It is plausible that individuals may choose or be constrained to a particular trip duration and distance and this drives variation in the way they search for food through the distribution of foraging patches. However, given wandering albatrosses forage on prey that have a patchy distribution, with prey often caught in different patches (Weimerskirch et al., 2007), the distance from the colony is unlikely to drive variation in prey availability. Moreover, as males and females show pronounced sexual segregation, the duration and distance of trips are unlikely to result in the same changes in prey distribution with space and time for both sexes.

At first glance, we would expect that if EE strategy drives differences in foraging trips, it would be more explorative individuals that would travel further, which is the opposite to the relationship presented here. However, in this study the EE trade‐off and exploration refers to the propensity to move between foraging patches, not the specific distances birds cover between these patches. What this means is these birds switch patches more frequently and have smaller patches. This could result in shorter distances travelled as if patches are small, and would decrease the length of the trip, if all other variables are equal. For example, if birds move the same distance between patches but one bird has much smaller patches, the cumulative distance will be reduced. Future work should focus on using behavioural models to identify step lengths between patches, which would allow the size of patch and the movement between them to be modelled simultaneously. This would reveal whether trips are shorter for bolder individuals as a result of the size of their patches and hence their EE strategy.

### 4.5 Habitat choice and the EE trade‐off

Given that boldness can affect foraging behaviour in certain age classes, foraging in different locations, and therefore habitats, may appear to be a plausible driver of differences in searching behaviour and hence EE strategy. Wandering albatrosses demonstrate little evidence of habitat preference nor strong responses to environmental heterogeneity, suggesting that small scale habitat cues and differences do not account for a large amount of individual variation in foraging behaviour (Louzao, Wiegand, Bartumeus, & Weimerskirch, 2014; Weimerskirch et al., 2007). Our results showing that EE strategy was not driven by sex provides further support that these differences are not as result of habitat. This is because the sexual segregation between males and females means they forage in very different areas with very different environmental conditions and habitats. If habitat was a main driver of EE strategy, we would expect a strong sex differences in the correlation structure among traits and future work should estimate individual metrics for the strength of the covariance between traits. However, to truly differentiate between environmental drivers and foraging decisions, individual oceanographic conditions should be compared against EE strategy to test whether the repeatability in this correlates with a repeatability in habitat choice. These results do, however, offer a proximate mechanism through which widely reported individual specialisation in foraging behaviour (Patrick et al., 2014) and diet in seabirds (Ceia & Ramos, 2015) can be explained.

### 4.6 Fitness consequences of EE strategy

We found no detectable fitness advantage of either strategy, nor any components of the EE trade‐off. As the power of the statistical test was low, we cannot exclude a very weak evolutive advantage of one strategy. Nevertheless, given that the number of patches and time in patches vary with age and sex, we could suggest that these traits decouple with the trade‐off when foraging success falls below a threshold. Individual birds should then increase effort to avoid deleterious effects on fitness, and this may explain strong links between age and sex on individual components. Fitness may not correlate with EE strategy, despite the correlation with trip length, if differences in trip duration are coupled with the frequency of trips. We were unable to test this in our study, as we had mostly single trips for an individual. However, as there is often a correlation in foraging trip duration between partners during incubation, an individual with short foraging trips may have a partner who displays the same behaviour and this would result in more frequent trips. While this suggests trip frequency could be another component of this trade‐off, the synchrony between pairs varies at the individual level, such that this would require additional data collection to test this hypothesis. Analyses should also be extended to examine lifetime reproductive success, the most accurate measure of fitness, and to test whether the correlation between traits breaks down as an individual shows declines in reproductive performance.

Recent theoretical models have demonstrated that individuals may differ in their switchover point between intensive (exploitation) and extensive (exploration) searching (Bartoń & Hovestadt, 2013), and these suggest that individual strategies can emerge as a result of differences in diet or prey distribution. Individual positions on the EE trade‐off may be explained if bold and shy individuals differ in their prey, not habitat choice. Given that we know that wandering albatrosses may catch several small prey in a row, or isolated larger prey (Weimerskirch, Cherel, Cuenot‐Chaillet, & Ridoux, 1997), individual searching strategies reported here may be driven by links between boldness and prey choice. This could be addressed in future studies by studying diet or using stable isotopes to identify broad prey types. Another explanation for the differences in EE strategy may be that as fast exploring (Cole & Quinn, 2011) and bolder individuals (Patrick & Weimerskirch, 2014) are often more competitive with other species, they may dominate smaller patches, or have higher foraging success, such that they require fewer foraging attempts to successfully obtain prey. However, wandering albatrosses experience little competition as they are solitary feeders and therefore are unlikely to be highly constrained in where they can search for food. Finally another aspect to explore in the future is the link with fishing boats: wandering albatrosses are known to be attracted by fishing boats (Collet, Patrick, & Weimerskirch, 2015), and differences in individual responses to these vessels due to personality may result in differences in the EE behaviour.

We know that foraging behaviour and the acquisition of resources is paramount to fitness and yet we lack a full understanding of the mechanisms through which individual foraging strategies emerge. Our results show individual suites of foraging traits form a trade‐off between exploration and exploitation. Moreover, we show that these differences appear to be stable and repeatable among individuals in the population, with no fitness implications, and are instead correlated with personality differences. These data highlight that changes from exploration to exploitation can be captured at the individual level and future work should focus on assessing the causes and consequences of switch points and how these strategies affect long‐term foraging effort and lifetime reproductive success.

## AUTHORS’ CONTRIBUTIONS

S.C.P. and H.W. conceived the ideas and designed methodology; S.C.P. and H.W. collected the data; S.C.P., D.P. and H.W. analysed the data; S.C.P, D.P. and H.W. contributed to the writing of the manuscript. All authors contributed critically to the drafts and gave final approval for publication.

## DATA ACCESSIBILITY

Data available from the Dryad Digital Repository: https://dx.doi.org/10.5061/dryad.k3b1m (Patrick, Pinaud, & Weimerskirch, 2017).
