# Supplementary material for: Boldness predicts an individual's position along an exploration–exploitation foraging trade‐off
Source: J Anim Ecol. 2017 Jul 24;86(5):1257–68. doi: 10.1111/1365-2656.12724 (PMC5601208; doi:10.1111/1365-2656.12724)
Supplement: Supplementary file 1 [file JANE-86-1257-s001.docx]

**Appendix 1: Data selection and model structure**

Appendix 1: Table S1: Details of the three datasets used the manuscript – Data (2) presented in the main paper and data (1) and data (3) shown in the supplementary material. The table outlines repeated measured per data set and hence random effects fitted in models. Full sample sizes for all analyses are also given. RS= reproductive success.

|  | *Data set 1* | *Data set 2* | *Data set 3* |
| --- | --- | --- | --- |
| *Time in patch, foraging effort* | Repeated measures per trip | Repeated measures per bird | Single measure per bird |
| *Size of patch, number of patches* | Repeated measures per bird | Repeated measures per bird | Single measure per bird |
| *Random effect (Tables 3 +4)* | Trip ID, Bird ID, Year | Bird ID, Year | Year |
| *Sample Sizes: Patches* | Total: 816  Known boldness + age + sex: 561  Known RS: 802 | Total: 292  Known boldness + age + sex: 197  Known RS: 282 | Total: 228  Known boldness + age + sex: 154  Known RS: 221 |
| *Sample Sizes: Trips* | Total: 276  Known boldness + age + sex: 193  Known RS: 269 | Total: 274  Known boldness + age + sex: 186  Known RS: 267 | Total: 228  Known boldness + age + sex: 154  Known RS: 221 |
| *Sample Sizes: Birds* | Total: 228  Known boldness + age + sex: 159  Known RS: 223 | Total: 228  Known boldness + age + sex: 155  Known RS: 223 | Total: 228  Known boldness + age + sex: 154  Known RS: 221 |

**Appendix 2: Results using different subsets of data**

Appendix 2: Table S1a: The correlations among foraging traits for all individuals in the population. Pearson’s correlation coefficients are shown on the top half of the matrix and p values on the bottom half. N patches = 816; N trips = 276; N birds = 228.

| *Foraging Variable* | *Time in patch* | *Foraging effort* | *Size of patch* | *Number of patches* |
| --- | --- | --- | --- | --- |
| *Time in patch* |  | **0.70** | **0.30** | **-0.09** |
| *Foraging effort* | **<0.001** |  | **0.23** | -0.06 |
| *Size of patch* | **<0.001** | **<0.001** |  | **-0.08** |
| *Number of patches* | **0.01** | 0.09 | **0.02** |  |

Appendix 2: Table S1b: The correlations among foraging traits for all individuals in the population. Pearson’s correlation coefficients are shown on the top half of the matrix and p values on the bottom half. Sample is restricted to one trip per bird across years, randomly selected. N patches = 228; N trips = 228; N birds = 228.

| *Foraging Variable* | *Time in patch* | *Foraging effort* | | *Size of patch* | | *Number of patches* |
| --- | --- | --- | --- | --- | --- | --- |
| *Time in patch* |  | **0.53** | **0.40** | | -0.07 | |
| *Foraging effort* | **<0.001** |  | **0.36** | | -0.05 | |
| *Size of patch* | **<0.001** | **<0.001** |  | | 0.06 | |
| *Number of patches* | 0.26 | 0.48 | 0.40 | |  | |

Appendix 2: Table S2a: Principal components (PC), weightings and variance explained from a principal components analysis. PC1 is used in the paper as a proxy for EE strategy. N patches = 816; N trips = 276; N birds = 228.

| *Foraging Variable* | *PC1* | *PC2* | *PC3* | *PC4* |
| --- | --- | --- | --- | --- |
| *Time in patch* | 0.65 | -0.13 | 0.21 | -0.72 |
| *Foraging effort* | 0.63 | -0.19 | 0.30 | 0.69 |
| *Size of patch* | 0.40 | -0.15 | -0.90 | 0.07 |
| *Number of patches* | -0.15 | -0.96 | -0.23 | -0.03 |
| *Proportion of variance explained* | 0.47 | 0.25 | 0.21 | 0.07 |
| *Cumulative variance explained* | 0.47 | 0.72 | 0.93 | 1.00 |

Appendix 2: Table S2b: Principal components (PC), weightings and variance explained from a principal components analysis. PC1 is used in the paper as a proxy for EE strategy. Sample is restricted to one trip per bird across years, randomly selected. N patches = 228; N trips = 228; N birds = 228.

| *Foraging Variable* | *PC1* | *PC2* | *PC3* | *PC4* |
| --- | --- | --- | --- | --- |
| *Time in patch* | 0.61 | -0.08 | 0.26 | -0.75 |
| *Foraging effort* | 0.59 | -0.05 | 0.47 | 0.65 |
| *Size of patch* | 0.53 | 0.24 | -0.81 | 0.13 |
| *Number of patches* | -0.05 | 0.97 | 0.24 | -0.06 |
| *Proportion of variance explained* | 0.47 | 0.26 | 0.16 | 0.12 |
| *Cumulative variance explained* | 0.47 | 0.72 | 0.88 | 1.00 |

Appendix 2: Table S3a: The relationship between boldness, age and sex with component traits and principal component one (EE strategy) of the EE trade-off. Estimates from general linear mixed models of slopes and intercepts are presented for significant effects. Non-significant interactions were dropped from all models and terms dropped from final models shown here in italics. N patches = 561; N trips = 193; N birds = 159.

|  |  | *Explanatory variables* | | | |
| --- | --- | --- | --- | --- | --- |
| *Response variable* | *Model output* | *Boldness* | *Age* | *Sex* | *Age x Sex* |
| *PC1 (*EE *strategy)* | Test statistic | χ^2^ _1_= 3.62; | χ^2^ _1_= 1.97; | χ^2^ _1_= 0.24; | *χ^2^ _1_= 0.44;* |
|  | P value | p = 0.06 | p = 0.16 | p = 0.62 | *p = 0.51* |
|  | *Estimate ± SE (logged scale)* | -0.07± 0.04 | 0.06± 0.04 | F:0.71 ± 0.12  M:0.75 ±0.12 | *-0.01± 0.01* |
| *Time in patch* | Test statistic | χ^2^ _1_= 2.38; | χ^2^ _1_= 2.21; | χ^2^ _1_= 0.10; | *χ^2^ _1_= 2.16;* |
|  | P value | p =0.12 | p = 0.14 | p = 0.76 | *p = 0.14* |
|  | *Estimate ± SE (logged scale)* | -0.07±0.04 | 0.07± 0.05 | F:-0.01± 0.15  M:0.01± 0.15 | *-0.15 ± 0.10* |
| *Foraging effort* | Test statistic | χ^2^ _1_ = 3.16; | χ^2^ _1_= 0.09; | χ^2^ _1_ = 0.35 | *χ^2^ _1_ = 0.02;* |
|  | P value | p = 0.08; | p = 0.77 | p = 0.56 | *p = 0.90* |
|  | *Estimate ± SE (logged scale)* | -0.08± 0.04 | -0.01± 0.05 | F:-0.16± 0.10  M:- 0.21± 0.10 | *0.01± 0.10* |
| *Size of patch* | Test statistic | NA | | | |
|  | P value |  |  |  |  |
|  | *Estimate ± SE (logged scale)* |  |  |  |  |
| *Number of patches* | Test statistic |  |  |  |  |
|  | P value |  |  |  |  |
|  | *Estimate ± SE (logged scale)* |  |  |  |  |

Appendix 2: Table S3b: The relationship between boldness, age and sex with component traits and principal component one (EE strategy) of the EE trade-off. Estimates from general linear mixed models of slopes and intercepts are presented for significant effects. Sample is restricted to one trip per bird across all years, randomly selected. N patches = 154; N trips = 154; N birds = 154.

|  |  | *Explanatory variables* | | | |
| --- | --- | --- | --- | --- | --- |
| *Response variable* | *Model output* | ***Boldness*** | ***Age*** | ***Sex*** | ***Age x Sex*** |
| *PC1 (*EE *strategy)* | Test statistic | **χ^2^ _1_=6.94;** |  |  | **χ^2^ _1_= 4.83;** |
|  | P value | **p = 0.008** |  |  | **p = 0.028** |
|  | *Estimate ± SE (logged scale)* | **-0.13± 0.05** |  |  | **-0.25± 0.11** |
| *Time in patch* | Test statistic | **χ^2^ _1_= 3.82;** |  |  | **χ^2^ _1_= 8.15;** |
|  | P value | **p =0.051** |  |  | **p = 0.004** |
|  | *Estimate ± SE (logged scale)* | **-0.12± 0.06** |  |  | **-0.43± 0.15** |
| *Foraging effort* | Test statistic | **χ^2^ _1_ = 5.08.** | χ^2^ _1_= 1.87; | χ^2^ _1_ = 0.01; | *χ^2^ _1_ = 2.11;* |
|  | P value | **p = 0.025;** | p = 0.17 | p = 0.92 | *p = 0.15* |
|  | *Estimate ± SE (logged scale)* | **-0.20± 0.093** | -0.13± 0.10 | F:-0.56± 0.13  M:-0.54± 0.12 | *-0.30± 0.02* |
| *Size of patch* | Test statistic | **χ^2^ _1_= 7.09;** | χ^2^ _1_= 0.72 | χ^2^ _1_= 2.21; | *χ^2^ _1_ = 0.51;* |
|  | P value | **p =0.008** | p = 0.40 | p = 0.14 | *p = 0.48* |
|  | *Estimate ± SE (logged scale)* | **-0.17± 0.06** | 0.06± 0.07 | F:-0.45± 0.13  M:-0.27± 0.13 | *-0.10± 0.14* |
| *Number of patches* | Test statistic | χ^2^ _1_=0.49; | χ^2^ _1_ = 0.77; | **χ^2^ = 2.91** | *χ^2^ = 0.35;* |
|  | P value | p = 0.49 | p = 0.38 | **p = 0.088** | *p = 0.56* |
|  | *Estimate ± SE (logged scale)* | -0.17± 0.06 | -0.04± 0.04 | **F:0.60± 0.08**  **M:0.47± 0.08** | *-0.05± 0.08* |

Appendix 2: Table S4a: Variance components (± SE) and repeatability (Confidence intervals) extracted from final models for foraging traits, boldness, sex and age (Table 3a). N patches = 561; N trips = 193; N birds = 159.

|  | *Random effect variance estimates* | | | | *Repeatability* | |
| --- | --- | --- | --- | --- | --- | --- |
| *Response variable* | *Trip ID* | *Bird ID* | *Year* | *Residual* | *Trip ID R (CI);*  *P value* | *Bird ID R(CI);*  *P value* |
| *PC1 (*EE *strategy)* | 0.14± 0.38 | 0.05± 0.21 | 0.07± 0.27 | 0.10± 0.31 | **0.40 (0.23, 0.59);**  **P < 0.001** | **0.19 (0, 0.30);**  **P = 0.045** |
| *Time in patch* | 0.19± 0.44 | 0.04 ± 0.19 | 0.13 ± 0.35 | 0.26 ± 0.51 | **0.30 (0.15, 0.46);**  **P < 0.001** | 0.07 (0, 0.23);  P = 0.115 |
| *Foraging effort* | 0.14±  0.38 | 0.04±  0.21 | 0.04± 0.19 | 0.35±  0.59 | **0.25 (0.10, 0.39);**  **P < 0.001** | 0.08 (0, 0.22);  P = 0.11 |
| *Size of patch* | NA | | | | | |
| *Number of patches* |  |  |  |  |  |  |

Appendix 2: Table S5a: The relationship between the EE strategy and foraging trip metrics. Estimates from general linear mixed models of slopes and intercepts are presented for significant effects. N patches = 816; N trips = 276; N birds = 228.

| *Response variable* | *Explanatory variables* | *Test statistic (df)* | *P value* | *Estimate ± SE (logged)* |
| --- | --- | --- | --- | --- |
| *EE strategy (PC1)* | *Duration (hours)* | **χ^2^ _1_ = 45.46** | **P < 0.001** | **0.20 ± 0.03** |
|  | *Total distance (km)* | **χ^2^ _1_ = 22.90** | **P < 0.001** | **0.14 ± 0.03** |
|  | *Maximum range (km)* | **χ^2^ _1_ = 13.16** | **P < 0.001** | **0.10 ± 0.03** |

Appendix 2: Table S5b: The relationship between the EE strategy and foraging trip metrics. Estimates from general linear mixed models of slopes and intercepts are presented for significant effects. Sample is restricted to one trip per bird across years, randomly selected. N patches = 228; N trips = 228; N birds = 228.

| *Response variable* | *Explanatory variable* | *Test statistic (df)* | *P value* | *Estimate* **±** *SE (logged)* |
| --- | --- | --- | --- | --- |
| *EE strategy (PC1)* | *Duration (hours)* | **χ^2^ _1_ = 26.72** | **P < 0.001** | **0.21 ± 0.04** |
|  | *Total distance (km)* | **χ^2^ _1_ = 20.88** | **P < 0.001** | **0.18 ± 0.04** |
|  | *Maximum range (km)* | **χ^2^ _1_ = 13.09** | **P < 0.001** | **0.14 ± 0.04** |

Appendix 2: Table S6a: The relationship between the EE strategy and individual components with reproductive success, estimated from general linear mixed models. N patches = 802; N trips = 269; N birds = 223

| *Response variable* | *Explanatory variables* | *Test statistic (df)* | *P value* | *Estimate ± SE (logged)* |
| --- | --- | --- | --- | --- |
| *Reproductive Success* | *PC1 (*EE *strategy)* | χ^2^ _1_ = 0.05 | P = 0.82 | 0.40 ± 1.83 |
|  | *Time in patch* | χ^2^ _1_ = 0.00 | P = 0.96 | 0.03 ± 0.65 |
|  | *Foraging effort* | χ^2^ _1_ = 0.40 | P = 0.53 | 0.49 ± 0.84 |
|  | *Size of patch* |  | | |
|  | *Number of patches* |  |  |  |

Appendix 2: Table S6b: The relationship between the EE strategy and individual components with reproductive success, estimated from general linear mixed models. Sample is restricted to one trip per bird, randomly selected. N patches = 221; N trips = 221; N birds = 221

| *Response variable* | *Explanatory variables* | *Test statistic (df)* | *P value* | *Estimate ± SE (logged)* |
| --- | --- | --- | --- | --- |
| *Reproductive Success* | *PC1 (*EE *strategy)* | χ^2^ _1_ = 0.21 | P = 0.64 | -0.18 ± 0.38 |
|  | *Time in patch* | χ^2^ _1_ = 0.36 | P = 0.55 | -0.10 ± 0.16 |
|  | *Foraging effort* | χ^2^ _1_ = 0.65 | P = 0.42 | -0.13 ± 0.16 |
|  | *Size of patch* | χ^2^ _1_ = 0.01 | P = 0.91 | -0.02 ± 0.16 |
|  | *Number of patches* | χ^2^ _1_ = 0.28 | P = 0.59 | 0.09 ± 0.17 |

**Figures**

Figure S1: Boldness and foraging traits: a) Boldness and principal component one, indicative of an individual’s position along an exploration exploitation trade-off. b) The size of foraging patches in relation to individual boldness. Bolder individuals have considerably smaller patches than shyer individuals.


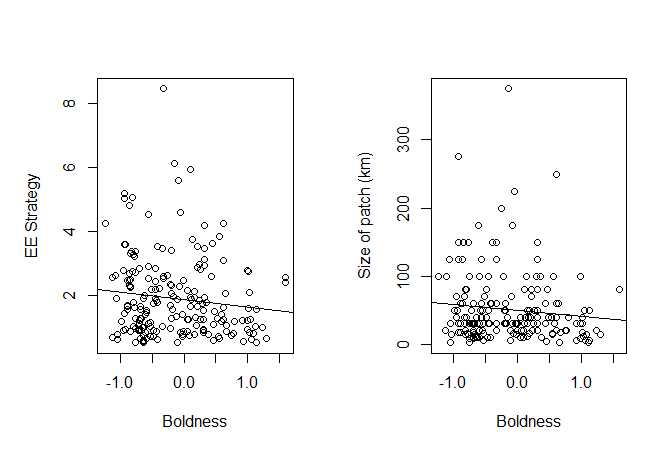


b)

a)

Figure S2: The relationship between EE strategy and foraging trip matrices. a) Individuals which favour exploitation have longer foraging trip durations. b) Individuals which favour exploitation travel further during trips. c) Individuals which favour exploitation have longer foraging trip durations.


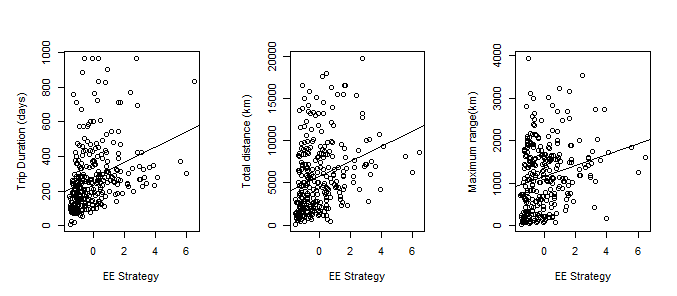


c)

b)

a)

**Appendix 3: Multivariate models**

**Methods**

We fitted multivariate mixed models in MCMCglmm (50) to examine the covariance between boldness, size of foraging patch, time in patch and foraging effort (56). We fitted boldness with an ordinal error distribution and included fixed effects for observation number, observer and age (48) and random effects for Individual ID. We fitted size of patch (Poisson error distribution), time in patch (Square root transformed Gaussian error distribution), foraging effort (Poisson error distribution) and number of patches (Poisson error distribution) with fixed effects for age and sex and random effects for year and, for time in patch and foraging effort, trip ID. We estimated the covariance between these five traits, while fixing the residual covariance to 1 and assessed the significance of all traits by examining the credibility intervals.

We selected our priors from current recommendations for models, using parameter expanded priors (de Villemereuil *et al*. 2013). The posterior distribution was sampled every 1000 iterations, following a burn in period of 100,000 iterations and a general run of 50,000,000 iterations). While models examining the covariation between foraging traits converged well, models estimating the covariation of these with boldness failed to fully converge. However, all results support those shown in the paper.

| **Trait** | Time in patch | Size of patch | Number of patches | Number of landings | Boldness |
| --- | --- | --- | --- | --- | --- |
| Time in patch |  | **0.71 (0.52 -0.88)** | -0.86 (-1.00-0.79) | **0.95 (0.83-0.99)** | -0.20 (-0.49 -0.07) |
| Size of patch |  |  | -0.64 (-0.85-0.70) | **0.67 (0.52- 0.80)** | **-0.21 (-0.40- -0.06)** |
| Number of patches |  |  |  | -0.90 (-1.00-0.78 | 0.11 (-0.64 -0.62) |
| Number of landings |  |  |  |  | -0.22 (-0.42-0.03) |
| Boldness |  |  |  |  |  |

Table S1: The covariance matrix from multivariate model, with the credibility intervals.
